# Role of mitochondria-bound HK2 in rheumatoid arthritis fibroblast-like synoviocytes

**DOI:** 10.3389/fimmu.2023.1103231

**Published:** 2023-07-17

**Authors:** Alyssa Torres, Sarah Kang, Christopher B. Mahony, Martha Cedeño, Patricia G. Oliveira, Marta Fernandez-Bustamante, Samuel Kemble, Teresina Laragione, Percio S. Gulko, Adam P. Croft, Elsa Sanchez-Lopez, Shigeki Miyamoto, Monica Guma

**Affiliations:** ^1^ Department of Medicine, University of California, San Diego, La Jolla, CA, United States; ^2^ Department of Orthopedic Surgery, University of California, San Diego, La Jolla, CA, United States; ^3^ Rheumatology Research Group, Institute of Inflammation and Ageing, Queen Elizabeth Hospital, University of Birmingham, Birmingham, United Kingdom; ^4^ Division of Rheumatology, Department of Medicine, Icahn School of Medicine at Mount Sinai, New York City, NY, United States; ^5^ Department of Pharmacology, University of California, San Diego, La Jolla, CA, United States

**Keywords:** rheumatoid arthritis, FLS, mitochondria, glucose metabolism, hexokinase

## Abstract

**Background:**

Glucose metabolism, specifically, hexokinase 2 (HK2), has a critical role in rheumatoid arthritis (RA) fibroblast-like synoviocyte (FLS) phenotype. HK2 localizes not only in the cytosol but also in the mitochondria, where it protects mitochondria against stress. We hypothesize that mitochondria-bound HK2 is a key regulator of RA FLS phenotype.

**Methods:**

HK2 localization was evaluated by confocal microscopy after FLS stimulation. RA FLSs were infected with Green fluorescent protein (GFP), full-length (FL)–HK2, or HK2 lacking its mitochondrial binding motif (HK2ΔN) expressing adenovirus (Ad). RA FLS was also incubated with methyl jasmonate (MJ; 2.5 mM), tofacitinib (1 µM), or methotrexate (1 µM). RA FLS was tested for migration and invasion and gene expression. Gene associations with HK2 expression were identified by examining single-cell RNA sequencing (scRNA-seq) data from murine models of arthritis. Mice were injected with K/BxN serum and given MJ. Ad-FLHK2 or Ad-HK2ΔN was injected into the knee of wild-type mice.

**Results:**

Cobalt chloride (CoCl_2_) and platelet-derived growth factor (PDGF) stimulation induced HK2 mitochondrial translocation. Overexpression of the HK2 mutant and MJ incubation reversed the invasive and migrative phenotype induced by FL-HK2 after PDGF stimulation, and MJ also decreased the expression of C-X-C Motif Chemokine Ligand 1 (CXCL1) and Collagen Type I Alpha 1 Chain (COL1A1). Of interest, tofacitinib but not methotrexate had an effect on HK2 dissociation from the mitochondria. In murine models, MJ treatment significantly decreased arthritis severity, whereas HK2FL was able to induce synovial hypertrophy as opposed to HK2ΔN.

**Conclusion:**

Our results suggest that mitochondrial HK2 regulates the aggressive phenotype of RA FLS. New therapeutic approaches to dissociate HK2 from mitochondria offer a safer approach than global glycolysis inhibition.

## Introduction

Hexokinases (HKs) catalyze the first committed step in glucose metabolism to yield glucose-6-phosphate (G6P) through phosphorylation (1). Although HKs are involved in the first irreversible step of glycolysis, a critical source of energy, they are strongly inhibited by G6P through product inhibition (1). Two of the more highly expressed HKs are isoforms HK1 and HK2. HK1 is constitutively expressed, and HK2 is not as expressed as HK1 besides insulin-sensitive tissues (1). Despite this, HK2 is induced in other tissues in various diseases and has been studied most thoroughly in several types of cancer (2–4). Upregulation of this particular isoform assists in relieving the increased need for nutrients from highly active cells for proliferation and other functions (5).

HK2 has an N-terminal domain that allows it to bind to the mitochondrial outer membrane voltage-dependent anion channel (VDAC) (6–8). Binding to VDAC enhances the affinity of HKs for ATP and makes them desensitized to inhibition by their product, G6P (9, 10). Mitochondrial HK2 protective effect is well established in the prevention of apoptosis by inhibiting Bax, a proapoptotic protein, and autophagy regulation (9, 10). Conversely, its dissociation triggers apoptosis (11–16). This dual role in glycolysis and cell protection makes HK2 an obvious target for several diseases as described by other groups (17–19).

Rheumatoid arthritis (RA) is a systemic autoimmune disease that mainly affects the synovial joints, leading to chronic inflammation, joint destruction, and loss of function (20, 21). The microenvironment in inflamed joints is characterized by hypoxia and low amount of nutrients as the hyperplastic intimal synovial lining forms an aggressive front and the distance between blood vessels and synoviocytes is increased (22–25). Many key signaling pathways that are activated by this microenvironment converge to adapt cell metabolism to support their growth and survival (26–28). For example, accelerated glucose metabolism to lactate is a hallmark of proliferative and activated cells and is an adaptation to the stressful and dynamic microenvironment of inflamed tissue (29–32).

Fibroblast-like synoviocytes (FLSs) are a key component of RA invasive synovium and display unique aggressive features, including increased migration, invasion, and proliferation (33–35). Among other pathogenic mechanisms, changes in FLS metabolism support FLS activation and aggressive phenotype (36). Our recent work and others have highlighted a critical role of glucose metabolism and, specifically, of HK2 in activated FLS and how it regulates FLS pathogenic functions (37–39).

Here, we test the hypothesis that mitochondria-bound HK2 is a key regulator of RA FLS phenotype. Our analysis demonstrates that full-length (FL)–HK2 overexpression is more effective at increasing the migration and invasion than HK2 without the mitochondrial binding motif. Dissociation of HK2 from mitochondria also decreased invasion and migration and made cells more prone to apoptosis. *In vivo* data also show that dissociation of HK2 from mitochondria in arthritic mouse models attenuates arthritis severity. Together, our results suggest that mitochondrial HK2 contributes to joint destruction in RA.

## Materials and methods

### Human FLS

Human FLSs were extracted from patients with RA undergoing total joint replacement. All patients with RA met the American College of Rheumatology 1987 revised criteria. FLSs were cultured in high-glucose Dulbecco's Modified Eagle Medium (DMEM) without pyruvate, supplemented with 10% fetal bovine serum (FBS), L-glutamine (8 mM; 4 mM for mice FLS), and penicillin/streptomycin (100 U/ml) (Gibco, ThermoFisher Scientific, Inc., Whaltham, MA).

### Cell lines and PBMC

Cell line Human embryonic kidney 293A (HEK293A) was purchased from ThermoFisher Scientific, Inc. (Whaltham, MA), and was cultured in high-glucose DMEM without pyruvate, supplemented with 10% FBS, L-glutamine (8 mM), and penicillin/streptomycin (100 U/ml) (Gibco, ThermoFisher Scientific, Inc., Whaltham, MA). Human peripheral blood mononuclear cells (PBMCs) were isolated from whole blood by Ficoll density gradient using Ficoll Plaque Premium (Sigma GE Healthcare, #17–544-02) or BD Vacutainer CPT Tubes (BD, #362753) as described somewhere else.

### Reagents

Platelet derived growth factor subunit BB (PDGF)-BB) was obtained from R&D Systems, Inc. (Minneapolis, MN). Methyl jasmonate (MJ), CoCl_2_, methotrexate (MTX), and tofacitinib (Tofa) were purchased from Sigma-Aldrich (Sant Louis, MO).

### Adenovirus infection

Ad-mHK2 FL, Ad-mHK2ΔN (N-terminal deletion mutant), Ad-15G (association to mitochondria peptide), and Ad-GFP were developed as previously described (40). Supernatants were collected and freeze-thawed three times for cell disruption and 0.22-µm–filtered for cell debris elimination. Titration of adenoviral vectors was assessed by cell lysis assay with HEK293A cells to obtain the multiplicity of infection (MOI) quantification. Overexpression experiments were performed by infecting FLS with 500 MOI. Experiments were performed 48 h after infection except specified otherwise.

### Western blot

FLSs were plated 100,000 cells per well and were disrupted in lysis buffer after treatment and collection. Proteins were separated by Sodium dodecyl-sulfate polyacrylamide gel electrophoresis (SDS-PAGE) and transferred to a nitrocellulose membrane through the semi-dry system (Invitrogen). After blocking for 1 h with 5% milk, blots were incubated overnight at 4**°**C with the following antibodies: rabbit anti-human/mouse HK2 at 1:500 (#2867, C64G5, Cell Signaling Technology, Danvers, MA), mouse anti-human HK2 at 1:500 (sc-374091, B8, Santa Cruz Co, Santa Clara, CA), mouse anti-human tubulin at 1:1,000 (Santa Cruz Co., Santa Clara, CA), rabbit anti-TOM20 at 1:500 (Cell Signaling Technology, Danvers, MA), and Glyceraldehyde-3-Phosphate Dehydrogenase (GAPDH) and tubulin (Santa Cruz Co., Santa Clara, CA). Horseradish peroxidase–conjugated anti-mouse Immunoglobulin G (IgG) or anti-rabbit IgG (Cell Signaling Technology, Danvers, MA) was used as secondary antibody at 1:2,000 dilution. Membranes were developed using a chemiluminescence system (Clarity Western ECL, BIORAD, Hercules, CA). Images were captured by the ChemiDoc™ XRS+ System (BIORAD, Hercules, CA).

### Mitochondrial fractionation

RA FLSs were plated to 70% confluence in two 143-cm^2^ plates per condition and stimulated according to the figure legends. Mitochondrial protein was isolated using a mitochondria isolation kit (#89801 from ThermoFisher Scientific). Mitochondrial fraction was lysed in 2% 3-[(3-cholamidopropyl)dimethylammonio]-1-propanesulfonate (CHAPS) in Tris-Buffered Saline (TBS) buffer and quantified using Bradford. All subsequent steps are according to the Western blot protocol mentioned above.

### Cytochrome C quantification

RA FLSs were plated 100,000 per well in a six-well plate. After treatment with MJ or vehicle, cells were collected, and cell fractions were obtained as above but diluted according to a cytochrome c quantikine ELISA kit (R&D Biosystems, #DCTC0), and ELISA was performed according to the manufacturer’s directions.

### HK activity assay

RA FLS and PBMCs were collected and diluted to 1 × 10^6^ cells/ml, and HK activity assay was performed according to the manufacturer’s instructions (Sigma-Aldrich, #MAK091).

### Confocal experiments

Cells (8 × 10^3^) per well of RA FLS were plated on Lab-Tek brand chamber slides (n = 3) and were either incubated with CoCl_2_ (150 µM), PDGF (20 ng/ml), MTX (1 µM), and Tofa (1 µM) or infected with Ad-GFP, Ad-HK2, and Ad-HK2ΔN. Cells were fixed with 4% formaldehyde, permeabilized with 0.1% Triton X-100, and then blocked with 1% donkey serum in Phosphate buffered saline (PBS) for 1 h at Reverse transcriptase (RT). Then, cells were stained with anti-ATP5B (MAB3494, Millipore) to visualize the mitochondria and anti-HK2 (NBP2-16814, Novus) in blocking solution overnight at +4°C, followed by anti-mouse Alexa Fluor 594 and anti-rabbit Alexa Fluor 488 for 1 h at RT. Nuclei were counterstained with 4',6-diamidino-2-phenylindole (DAPI) and mounted with Fluosave. Cells were imaged at ×40 magnification using a DM6000 Leica microscope, images were analyzed, and HK2 colocation in the mitochondria was quantified using ImageJ Fiji software.

### Invasion

One million FLSs were trypsinized with 0.05% trypsin (Gibco, ThermoFisher Scientific, Inc., Whaltham, MA) and resuspended in 40 µl of 1% FBS DMEM and 40 µl of Matrigel (356231, BD Biosciences, San Diego, CA). Then, 4-µl drops of the mixture were plated in a six-well plate. After 5 min of incubation at 37**°**C, medium was added in the presence or absence of PDGF (10 ng/ml). The assay was stopped 24 h later, by adding 4% paraformaldehyde (PFA). Cells were stained with 0.05% crystal violet (Sigma-Aldrich, San Luis, MO), and images were captured with a 4× objective inverted microscope. For quantification, four images were taken per condition, and invasive area was measured by ImageJ software using arbitrary units.

### Migration

FLSs were seeded onto six-well plates and allowed to come to confluence. A double–cross-scratch wound was done in each well with the end of a sterile pipette tip. Cells were subsequently exposed to 1% FBS DMEM in the presence or absence of PDGF (10 ng/ml) for 24 h. FLSs were fixed by adding 4% PFA. Crystal violet was used at a concentration of 0.05% in distilled water for 30 min. After staining, cells were washed three times with water, and FLS migration across the wound margins was assessed, photographed, and measured by ImageJ—the average of relative length between random spots in all eight arms of the two cross sections.

### Death assay

Cells were plated at 50,000 cells per well in a 12-well plate. Cells were starved in 0.1% medium and, the following day, given MJ (1 µM) for 1 h before adding H_2_O_2_ to final concentrations of 75 or 200 µM. After 4 h, cells were fixed by adding 4% PFA. Cells were stained with 0.05% crystal violet (Sigma-Aldrich, San Luis, MO). Images were captured with a 10× objective inverted microscope. For quantification, mean gray value was measured through ImageJ software as described (41).

### Real-time PCR

FLSs were plated at 100,000 cells per well in a six-well plate using three-well replicates per condition. Cells were allowed to adhere overnight and then incubated 6 h with either ethanol (EtOH, vehicle) or MJ (1.5 mM). After the incubation time, cells were collected with TRIzol reagent (Invitrogen, 15596026). RNA was then extracted using chloroform, isopropyl alcohol, and 70% ethanol before adding 500 ng of each sample to do a reverse transcriptase reaction with the Bio-Rad iScript cDNA synthesis kit #1708891. The complementary DNA (cDNA) samples were then ran for real-time polymerase chain reaction (PCR) using primers from Integrated DNA technologies (IDT) and SsoAdvanced Universal SYBR green super mix from Bio-Rad Laboratories, Inc. Plates were analyzed with a Thermo Fisher QuantStudio 3 Real-Time PCR Systems machine. Relative mRNA was calculated in comparison with 18S gene as housekeeping. Primer sequences are available upon request.

### MTT viability assay

FLSs were plated at 3,000 cells per well in a 96-well plate using three-well replicates per condition per cell line. Conditions were added comparing 1% and 10% FBS. After 24 h with treatment, 10% 3-(4,5-dimethylthiazol-2-yl)-2,5-diphenyltetrazolium bromide (MTT) was added for 4 h and dissolved in 200 µl of Dimethyl Sulfoxide (DMSO). Optical density was measured at 550 and 690 nm, and replicates were averaged per condition and cell line.

### Single-cell RNAseq

The C57BL/6 mice were obtained from Charles River. All mice utilized for experimental procedures were 8- to 10-week-old male mice. Single animals were regarded as experimental entities. Induction of serum transfer-induced arthritis (STIA) was done by intravenous injection consisting of 100 μl of serum derived from KRN mice (K/BxN). Bones with intact joints from hind limbs of day 0, peak days 7–9, resolving day 15, or resolved day 22 and STIA inflamed mouse joints (*n* = 3 biological replicate samples, each consisted of cells isolated from the joints of three animals) were dissected and transferred into RPMI-1640 (+2% Fetal calf serum (FCS)) containing collagenase D (0.1 g/ml; Roche) and DNase I (0.01 g/ml; Sigma-Aldrich). Samples were incubated at 37°C for 40 min, followed by incubation with medium containing collagenase dispase (0.1 g/ml; Roche) and Deoxyribonuclease I (DNase I) (0.01 g/ml) at 37°C for 20 min. Live CD45^−^ synovial cells were sort purified using the BD FACSaria and captured with the 10x Genomics Chromium system. Sequencing libraries were generated using the 10x Genomics Single Cell 3′ Solution (version 2) kit and subjected to Illumina sequencing (HiSeq 4000, read 2 sequenced to 75 bp). Alignment to mm10 was completed using CellRanger (10x Genomics, v2.1.1). Analysis was completed using and R (v4.1) and using Seurat (v4.0.3) (42). Data were then analyzed in Seurat, where the following quality control (QC) metrics were used for all samples: nFeature_RNA > 500, nFeature_RNA <5,000, nCount_RNA > 500, and nCount_RNA < 20,000 (except PEAK_A, PEAK_B, PEAK_C, RESING_A, RESING_B, and RESING_C, where the maximum nCount_RNA was 40,000, 40,000, 40,000, 25,000, 30,000, and 30,000, respectively). Differential expression between clusters was calculated using FindAllMArkers() and FindMarkers() (between time points) in Seurat. Gene ontology (GO) term analysis was completed using gsFisher (v0.2), and Volcano plots were plotted using EnahcedVolcano (v1.13.2) R packages. Murine data are available at GSE230145.

### Mice

KRN T cell receptor–transgenic mice were a gift from Drs. D. Mathis (Harvard Medical School, Boston, MA) and C. Benoist (Institut de Génétique et de Biologie Moléculaire et Cellulaire, Strasbourg, France). All mice used in these experiments were bred on the C57BL/6 background and were 8–12 weeks old. All protocols involving animals received prior approval from the Institutional Review Boards and followed the Guide for the Care and Use of Laboratory Animals from the Institute for Laboratory Animal Research (National Research Council).

### K/BxN passive induce arthritis

Sera from adult, arthritic K/BxN mice were pooled for use in serum transfer. Recipient mice were injected intraperitoneally with 150 µl of the K/BxN mouse serum. One hour before serum injection (d0) or 5 days after serum injection (d5), MJ or vehicle was injected into the mouse at either 25 or 100 mg/kg. Clinical arthritis scores were evaluated in the recipient mice after serum transfer, as described previously (43). On day 12 after serum injection, the mice were euthanized, and their paws were analyzed for histopathological changes.

### Adenovirus mice injection

A total of 1 × 10^10^ viral particles in 8 µl were injected in the knees of control C57BL/6 mice. Ad-HK2FL was injected in the right knee, whereas Ad-HK2ΔN was injected in the left knee. Fourteen days after the injection, mice were euthanized, and knees were collected for histological studies and quantified as below.

### Antigen-induced arthritis induction

Experimental antigen-induced arthritis (AIA) was induced by a subcutaneous injection of 100 μg of methylated bovine serum albumin (mBSA) emulsified in 100 μl of complete Freund’s adjuvant (CFA) in the flank and, 1 week later, by an intradermal injection of 100 μg of mBSA/CFA in the tail base. Two weeks after these injections, arthritis was induced by intraarticular injection of 60 μg of mBSA in 10 μl of saline into the knee joint. After 3 days, a total of 1 × 10^10^ viral particles in 8 µl of Ad-GFP and Ad-15G were administered with intraarticular injection in the knees. Disease was assessed 14 days after intraarticular injection (11 days after adenovirus injection) by histological analysis as described below.

### Histology analysis

Joints were fixed in 10% formalin, decalcified in 10% EDTA for 2–3 weeks, and paraffin-embedded. Paraffin-embedded human synovium and mice joints sections were stained with hematoxylin and eosin and safranin O (43). A blinded semiquantitative scoring system was used to assess synovial inflammation, synovial hypertrophy, bone erosion, and cartilage damage (scale of 0–5) as previously described (43).

### Statistical analysis

Statistical analysis was performed with Prism software (version 5; GraphPad). Results are expressed as the mean ± SEM. Normality of the variables was assessed using the Kolmogorov–Smirnov and D’Agostino–Pearson normality tests. For comparison between two groups, Student’s two-tailed unpaired *t*-tests or nonparametric Mann–Whitney tests were applied, depending on the normality of the distribution of the variables. We compared three or more groups with analysis of variance (ANOVA) using Kruskal–Wallis non-parametric test followed by Dunnett’s *post-hoc* test or with one-way ANOVA parametric test followed by Tukey’s *post-hoc* test, depending on the homogeneity of variances. Results were considered significant if the two-sided *P*-value was less than 0.05. In all the figures, * is p<0.05, ** is p <0.01, *** is p <0.005, **** is p<0.001.

## Results

### Confocal microscopy and cell fractionation indicate that stimulation with CoCl_2_ and PDGF induces HK2 localization to mitochondria

**Figure 1:**
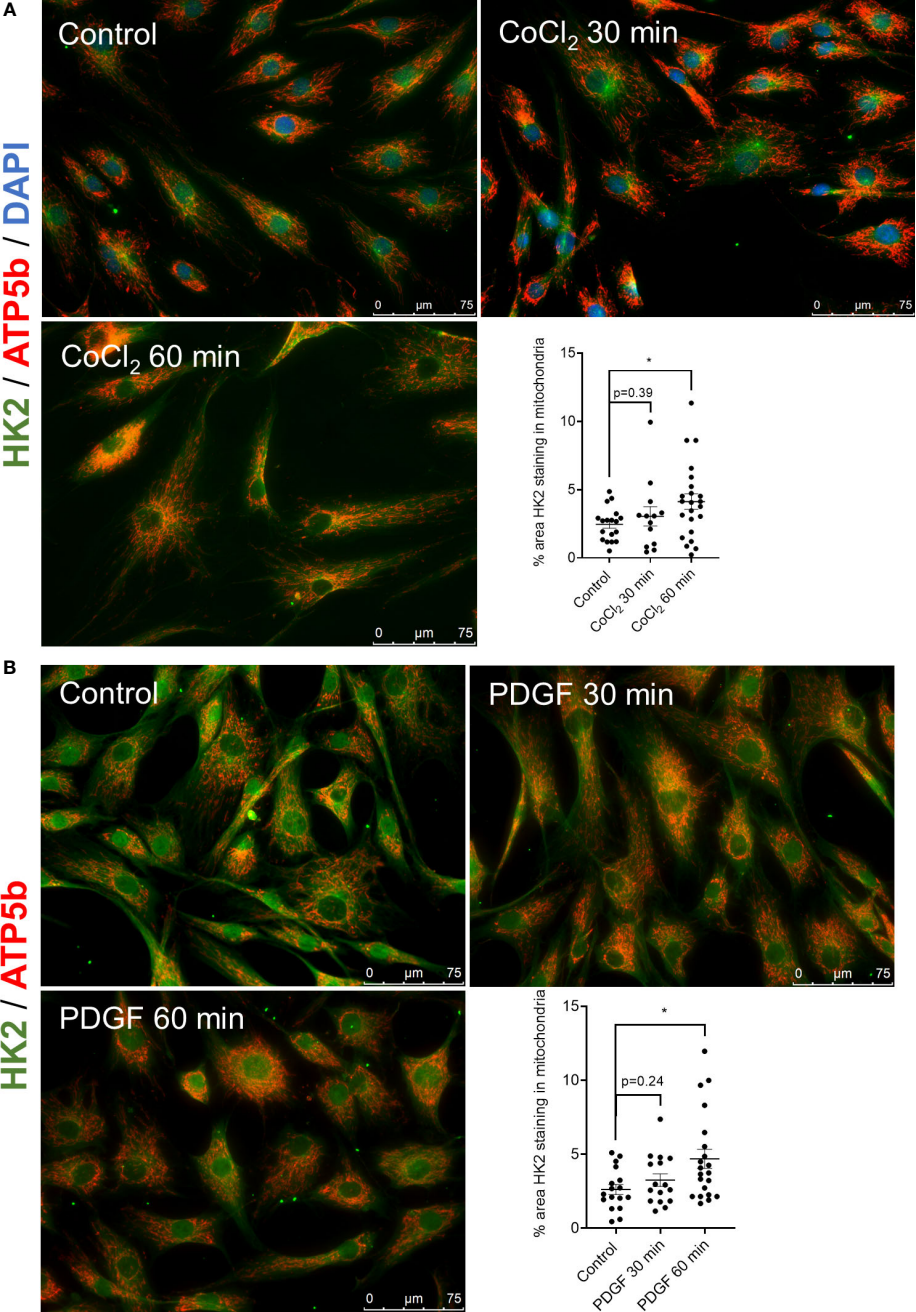
HK2 translocates to mitochondria after CoCl_2_ and PDGF stimulation. **(A)** Confocal microscopy of RA FLS (n = 3) after stimulation with 150 µM cobalt chloride (CoCl_2_) for 30 and 60 min with quantification. HK2 protein is stained with green fluorescence, ATP5a is stained with red fluorescence, and DAPI is stained with blue fluorescence as control. Overlapping yellow color indicates HK2 colocalization to mitochondria. n = 3 independent experiments. The comparison between groups was performed using unpaired two-tailed Student’s *t*-test. Statistical significance was considered when p-value ≤ 0.05. **(B)** Confocal microscopy of RA FLS (n = 3) after stimulation of PDGF (20 ng/ml) after 30 and 60 min with quantification. HK2 protein is stained with green fluorescence and ATP5a is stained with red fluorescence. Overlapping yellow color indicates HK2 colocalization to mitochondria. n = 2 independent experiments. The comparison between groups was performed using unpaired two-tailed student *t*-test. Statistical significance was considered when p-value ≤ 0.05. *P ≤ 0.05.

We first determine whether HK2 binds to the mitochondria after stimulation with inflammatory mediators implicated in FLS responses. Stimulation of RA FLS with CoCl_2_, which mimics hypoxia, and PDGF for 60 min triggered HK2 mitochondrial binding but not its HK activity (**Supplementary Figure 1A**), as detected by an overlap of HK2 in green and ATP5b (a marker of mitochondria) in red by immunofluorescent confocal microscopy (**Figures 1A, B**). Binding of HK2 to mitochondria is critical for RA FLS aggressive behavior

**Figure 2:**
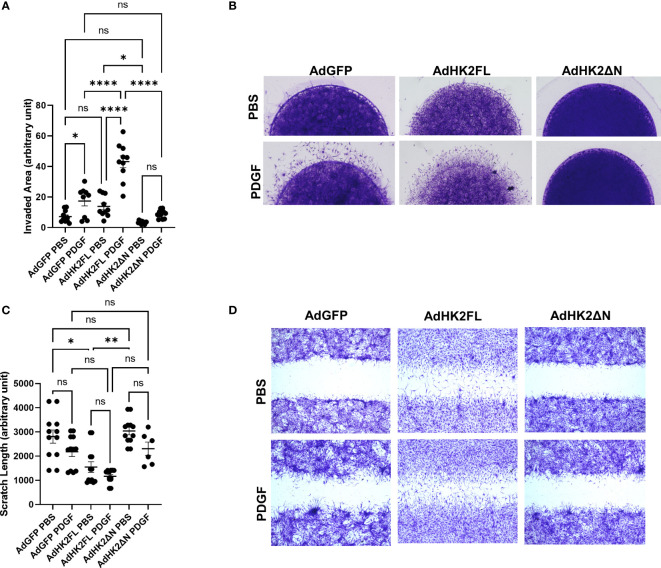
Effect of mitochondrial mutant HK2 on RA FLS invasion and migration. **(A, B)** RA FLSs were plated in matrigel spheroids as described in methods after adenovirus infection with Ad-GFP control, Ad-HK2FL, and Ad- HK2ΔN constructs for 2 days. **(A)** Analysis of area of invasion after infection with adenovirus. The comparison between groups was performed using ordinary one-way ANOVA followed by Tukey’s multiple comparisons test. Statistical analysis of P-value ≤ 0.05 was considered significant. **(B)** Representative images of invasion with the adenovirus constructs with and without PDGF. **(C, D)** RA FLSs were plated in six-well plates and scratched with the end of a sterile pipette tip after infection with adenovirus constructs. Cells were left to migrate 24 h with and without PDGF (10 ng/ml) stimulation. **(C)** Quantification of scratch length. The comparison between groups was performed using Kruskal–Wallis test followed by Dunn’s multiple comparisons test. Statistical significance was considered when p-value ≤ 0.05. **(D)** Representative images of migration with different adenovirus constructs with and without PDGF stimulation. ****P ≤ 0.001; **P ≤ 0.01. "ns" denotes "not significant".

HK2 has motif at the N-terminal that allows binding to mitochondria (schematic representation in **Supplementary Figure 1B**). We determined the role of the binding motif in FLS phenotype, by transducing the FLS with adenovirus carrying FL murine HK2, Ad-HK2 (FL), which has both kinase activity and mitochondrial binding motifs, and a truncated version of HK2, Ad-HK2ΔN, which has kinase activity but lacks of mitochondrial binding motif, which prevents HK2 from binding to the mitochondria. Adenovirus with GFP was used as control. **Supplementary Figure 1C** exhibits the upregulation of HK2, but not endogenous HK2, protein after infection with Ad-HK2FL and Ad-HK2ΔN. On the other hand, confocal immunofluorescent studies (**Supplementary Figure 1D**) indicate that there is a significant binding of HK2 to the mitochondria when incubated with Ad-HK2FL adenovirus rather than Ad-HK2ΔN adenovirus. **Figures 2A-D** demonstrate the potential role of HK2FL in aggressive behavior of RA FLS by looking at invasion (**Figures 2A, B**) and migration (**Figures 2C, D**). RA FLS invaded and migrated after infection with Ad-HK2FL and PDGF stimulation in comparison with GFP. This phenotype was not observed after the infection with Ad-HK2ΔN, suggesting that HK2 binding to the mitochondria plays a role in RA FLS aggressive phenotype.

### Treatment with MJ reduced RA FLS invasion and migration and induced apoptosis

Several drugs have shown to dissociate HK2 from mitochondria (44–46); among them, MJ is a plant stress hormone shown to dissociate HK2 from mitochondria in cancer cells as well as suppress proliferation and induce cell death (47, 48). Results from MTT viability assays revealed that MJ was nontoxic to RA FLS at a wide range of concentrations (**Supplementary Figure 2A**). In addition, MJ did not lead to a significant dissociation of cytochrome c from the mitochondria to the cytoplasm, which would be an indication of apoptosis (**Supplementary Figure 2B**). Mitochondrial fractionation protein analysis shows that HK2 expression in the mitochondrial fraction decreased after 60 min of incubation with MJ (**Supplementary Figure 2C**), which indicates MJ’s potential for dissociating HK2 from the mitochondria.

**Figure 3:**
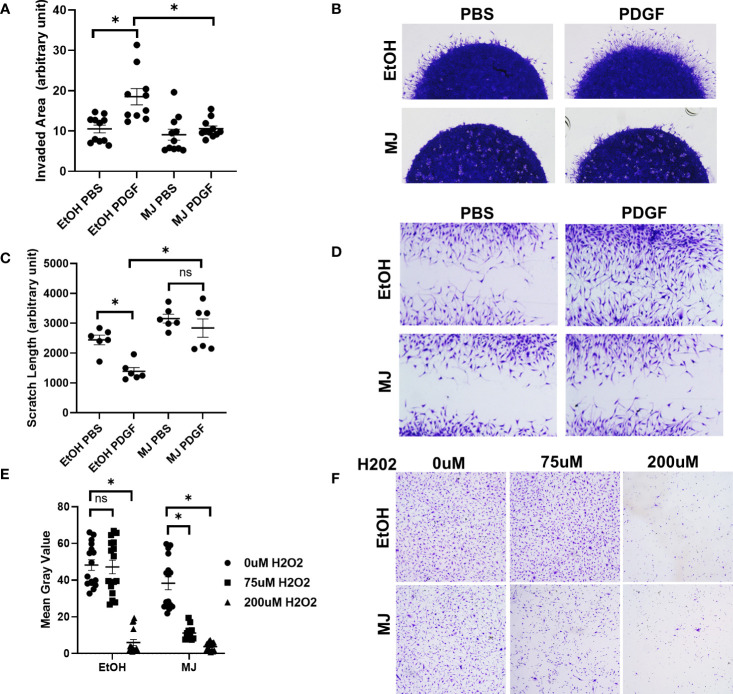
Treatment with methyl jasmonate (MJ), which dissociates HK2 from mitochondria, impaired FLS invasion and migration. **(A, B)** RA FLSs (n = 4) were plated in matrigel spheroids and given vehicle (ethanol) or MJ 1 h before PDGF stimulation. **(A)** Quantification of area of invasion after MJ treatment. Comparison between groups was performed using Kruskal–Wallis test followed by Dunn’s multiple comparisons test. Statistical analysis of P-value ≤ 0.05 was considered significant. **(B)** Representative images of invasion with MJ. **(C, D)** RA FLSs were plated in six-well plates and scratched with the end of a sterile pipette tip. Cells were given vehicle (ethanol) or MJ 1 h before PDGF stimulation and allowed to migrate for 24 h, **(C)** Quantification of scratch length. The comparison between groups was performed using ordinary one-way ANOVA followed by Tukey’s multiple comparisons test. Statistical analysis of P-value ≤ 0.05 was considered significant. **(D)** Representative images of MJ migration. **(E, F)** RA FLSs were plated in 12-well plates and treated with vehicle (ethanol) or MJ 1 h before addition of 0 µM H_2_O_2_, 75 µM H_2_O_2_, and 200 µM H_2_O_2_ to assess cell death and were fixed and stained after 4 h, **(E)** Analysis of mean gray value of cells using Fiji software. Comparison between groups was performed using Kruskal–Wallis test followed by Dunn’s multiple comparisons test. Statistical analysis of P-value ≤ 0.05 was considered significant. **(F)** Representative images of control and 1.5 mM MJ. *P ≤ 0.05. "ns" denotes "not significant".

We next looked at the effect of MJ on invasion and migration, two important characteristics in RA FLS phenotype. In RA FLS, MJ significantly reduced both RA FLS invasion and migration after PDGF stimulation (**Figures 3A-D**). Because RA FLSs are also known to be resistant to apoptosis, we then conducted a death assay to determine the RA FLS resistance after addition of MJ (**Figures 3E, F**). MJ was found to potentiate H_2_O_2_-induced apoptotic cell death in comparison to EtOH control.

### Effect of common RA drugs and inhibitors on HK2 translocation

**Figure 4:**
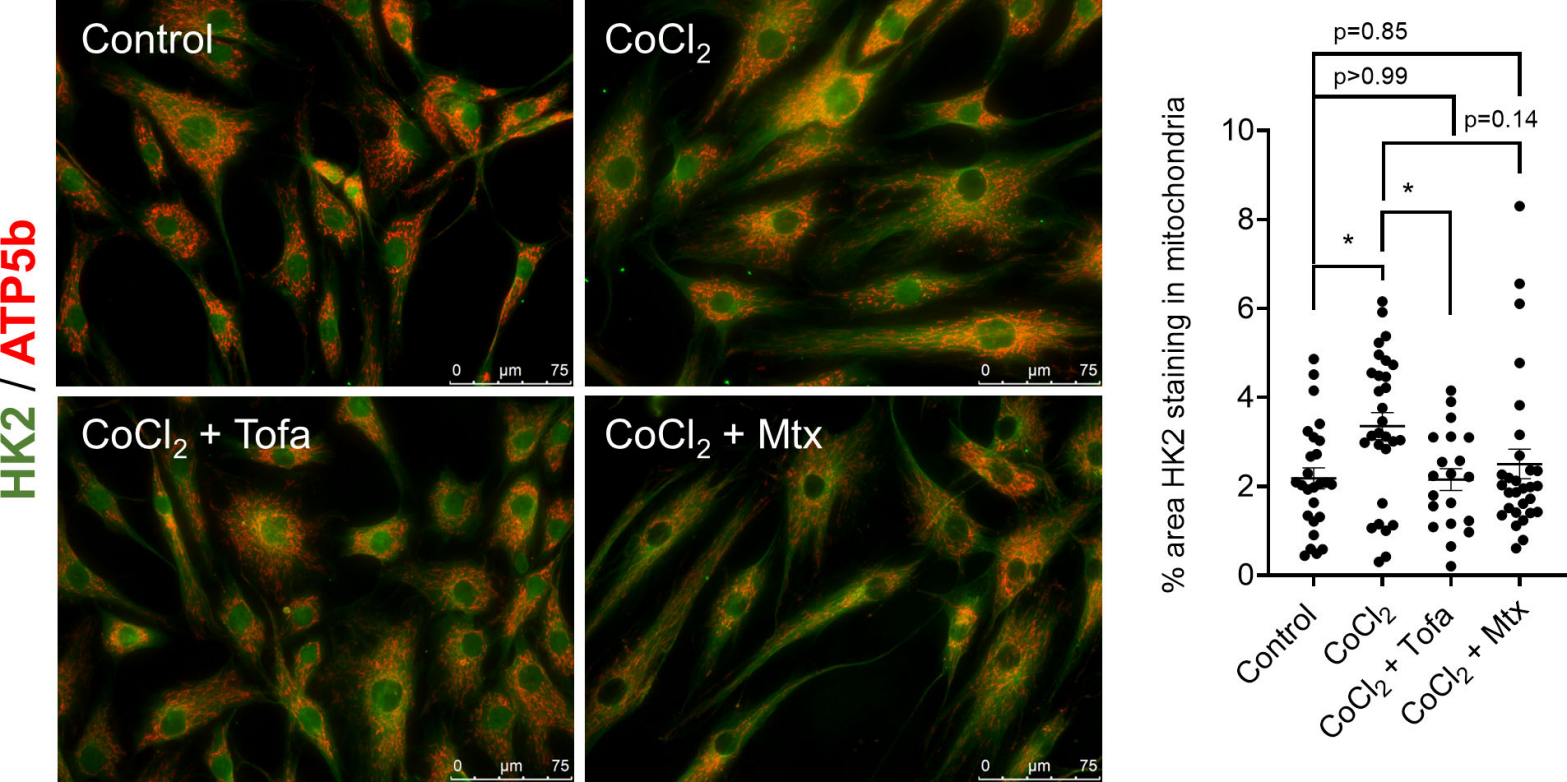
Effect of common RA drugs on mitochondrial translocation of HK2. Confocal microscopy of RA FLS (n = 3) after 60 min of stimulation of 150 µM CoCl_2_ with and without either 1 µM tofacitinib for 60 min or 1 µM MTX overnight with quantification. HK2 protein is stained with green fluorescence, and ATP5a is stained with red fluorescence. Overlapping yellow color indicates HK2 colocalization to mitochondria. n = 3 independent experiments. The comparison between groups was performed using ordinary one-way ANOVA followed by Tukey’s multiple comparisons test. Statistical analysis of P-value ≤ 0.05 was considered significant. *P ≤ 0.05.

We also determined HK2 adherence to the mitochondria under the presence of approved drugs in the treatment of RA, which include MTX and Tofa. Using immunofluorescent confocal microscopy, the addition of Tofa but not MTX decreased the binding of HK2 to the mitochondria after CoCl_2_ stimulation (**Figure 4**). However, Tofa was not able to avoid the HK2 translocation after adenovirus overexpression of HK2 (**Supplementary Figure 3**).

### MJ decreased the expression of genes associated to HK2 identified by scRNA-seq

**Figure 5:**
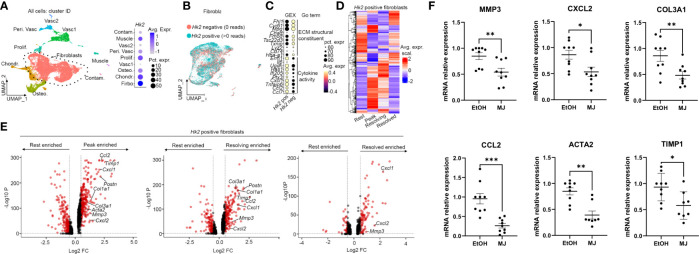
MJ decreased the expression of genes associated to HK2 identified by scRNA-seq. **(A)** UMAP of scRNAseq data of live CD45^−^ cells from digested hind limbs with main cell labels (n = 3 mice). Vasc, vascular cells; Peri. Vasc, perivascular; Contam., contamination; Osteo., osteoblasts; Chondr., chondrocytes. **(B)** Fibroblast subsets with cell labels based on reads of *Hk2* gene. **(C)** Gene expression (GEX) of top 10 marker genes in Hk2-positive cells compared with *Hk2* -egative cells with GO term. **(D)** Heatmap of differential expressed genes between disease model time point (rest, resolved, peak, and resolving) in *Hk2*-positive cells. **(E)** Volcano plots of differentially expressed genes between the indicated conditional in *Hk2*-positive cells. Each dot represents a gene. Genes in red: adjusted p-value < 0.05 and fold change > 0.5 and < −0.5. P-value calculated using FindMarkers() (Seurat) and the Wilcox method. **(F)** qPCR analysis of the indicated genes in RA FLS 6 h of MJ treatment. Results are average of three different RA FLS lines. Statistical analysis of P-value ≤ 0.05 was considered significant. *P ≤ 0.05 ; **P ≤ 0.01; ***P ≤ 0.001.

We next identified genes associated to HK2 synovial fibroblasts by examining scRNA-seq data from the STIA model. We first digested synovial tissue from hind limbs from mice at different time points of the disease stage (rest day 0, peak days 7–9, resolving day 15, and resolved day 22; **Supplementary Figure 4A**). Live CD45^−^ synovial cells were sort purified. 3′ mRNA sequencing generated 20,216 cells that passed QC (**Figure 5B**, **Supplementary Figure 4B**). We then annotated the main cell types (**Figure 5A**) based on marker gene expression (**Supplementary Figures 4C–H**) and subset fibroblasts (**Figure 5B**, **Supplementary Figure 4I**), which included the lining layer (**Supplementary Figure 4K**). Of interest, fibroblasts were the CD45^−^ synovial cells with higher *Hk2* expression (**Figure 5A**). We then selected *Hk2* positive (cell with >0 *Hk2* gene counts) and *Hk2* negative (cells with 0 *Hk2* gene counts) and examined marker gene expression. We found that *Hk2*-positive cells were enriched in pro-inflammatory molecules when compared with *Hk2*-negative cells (**Figure 5C**, **Supplementary Figure 4J**). We then examined differential gene expression across disease time points in *Hk2*-positive fibroblast and found a number of genes enriched in each time point when compared with rest (**Figure 5D**). Further examination of the differentially expressed genes revealed an upregulation of key inflammatory and matrix remodeling genes at peak (*Ccl2*, *Timp1*, *Cxcl1*, *Postn*, *Col1a1*, *Col3a1*, *Acta2*, *Mmp3*, and *Cxcl2:* **Figure 5E**). Many of these genes retained their upregulation in resolving, and most were not significantly differentially expressed in the resolved time point compared with rest (**Figure 5E**). We then sought to validate the role of the mitochondrial binding in the upregulation of these genes in human RA FLS. MJ decreased the expression of several of the genes associated to HK2 expression (**Figure 5F**).

### Truncated HK2 overexpression and HK2 dissociation with from mitochondria Ad-15G injection lead to a reduced synovial lining hypertrophy in murine healthy joints

**Figure 6:**
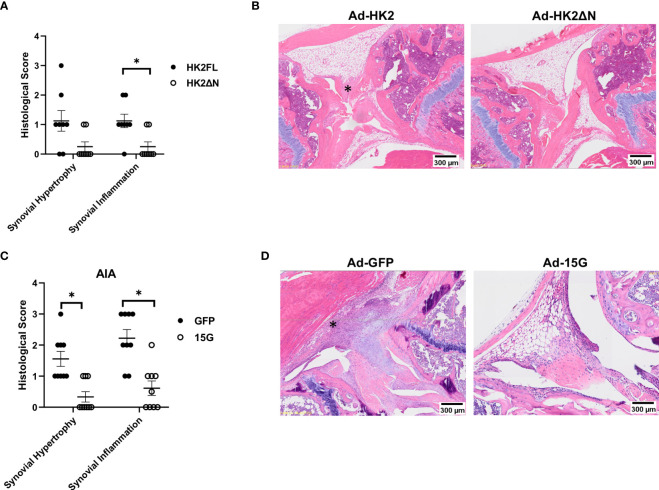
Adenovirus injection and Ad-15G treatment in AIA model. **(A, B)** B6 mice (n = 10) injected with AdenoHK2FL or Adeno HK2ΔN in either knee as described in methods. **(A)** Histological scores of mouse knees measuring synovial infiltration and infiltration of either HK2FL or HK2ΔN. The comparison between groups was performed using two-tailed Mann–Whitney non-parametric test for each score type. Statistical significance was considered when p-value ≤ 0.05. **(B)** Representative images of knee joints of mice. Asterisk (*) shows synovium **(C, D)** AIA mouse model (n = 10) with treatment of 15G peptide that dissociates HK2 from mitochondria as described in methods. **(C)** Histological score of mouse knees measuring synovial infiltration or infiltration of mice injected with Ad-GFP control or Ad-15G. The comparison between groups was performed using two-tailed Mann–Whitney non-parametric test for each score type. Statistical significance was considered when p-value ≤ 0.05. **(D)** Representative images of mouse joints.

We finally determined the effect of HK2 with and without binding motif on arthritis in experiments *in vivo*. Adenovirus injection was given on each knee of a C57BL/6 mouse, with Ad-HK2FL on one knee and Ad-HK2ΔN into the other knee (**Figures 6A, B**). After injection with adenovirus particles, mice were euthanized after 14 days and analyzed with histological staining. Knees injected with Ad-FLHK2 had higher synovial hypertrophy and inflammation scores as compared with the Ad-ΔNHK2 (**Figures 6A, B**).

Selective dissociation of HK2 from mitochondria was achieved using Ad-15G, an adenovirus encoding the first 15 amino acids of the mitochondrial binding motif (schematic is displayed in **Supplementary Figure 1A**). This competitively inhibits endogenous HK2 binding to mitochondria (40). In an AIA model of arthritis, the injection of Ad-15G reduced histological scores compared with Ad-GFP injection (**Figures 6C, D**).

### MJ treatment that dissociated HK2 from mitochondria significantly decreased arthritis severity in mice

**Figure 7:**
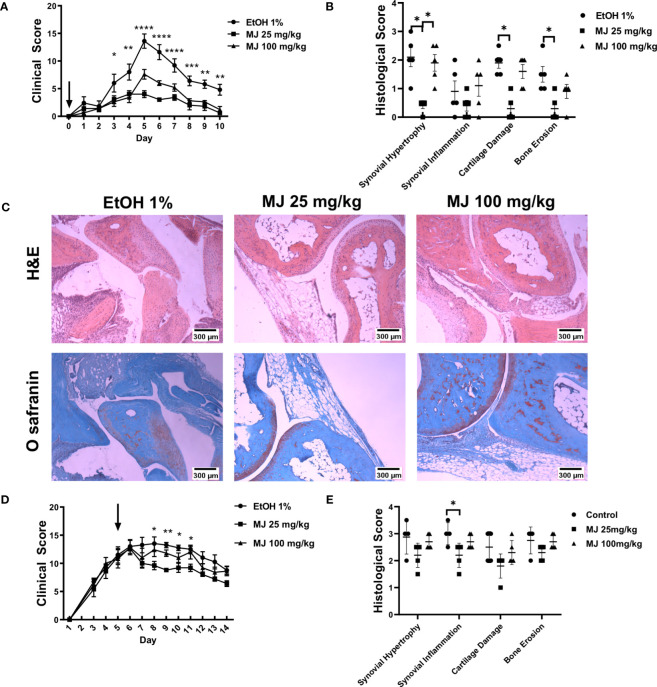
MJ treatment ameliorates arthritis in an animal model of inflammatory arthritis. B6 mice (n = 5) were injected with 150 µl of K/BxN serum. **(A)** Clinical score of mice after given MJ (25 or 100 mg/kg) same day of serum injection. Arrow points at day of MJ treatment. The comparison between groups was performed using ordinary one-way ANOVA followed by Tukey’s multiple comparisons test for clinical score. Statistical analysis of P-value ≤ 0.05 was considered significant. **(B)** Histological score of mice knee joints measuring synovial infiltration, infiltration, cartilage damage, and bone damage. Results of clinical and histological scores are average of five mice and graphed with SEM. The comparison between groups was performed using Kruskal–Wallis test followed by Dunn’s multiple comparisons test for each score type. Statistical analysis of P-value ≤ 0.05 was considered significant. **(C)** Images of hematoxylin and eosin staining and Safranin O staining with and without treatments of MJ. **(D)** Clinical score and joint diameter of mice after given MJ (25 or 100 mg/kg) on day 5 after serum injection. Arrow points at day of MJ treatment. The comparison between groups was performed using ordinary one-way ANOVA followed by Tukey’s multiple comparisons test for clinical score. Statistical analysis of P-value ≤ 0.05 was considered significant. **(E)** Histological scores of mice knee joints measuring synovial infiltration, infiltration, cartilage damage, and bone damage. Results of clinical and histological scores are average of five mice and graphed with SEM. The comparison between groups was performed using Kruskal–Wallis test followed by Dunn’s multiple comparisons test for each score type. Statistical analysis of P-value ≤ 0.05 was considered significant. *P ≤ 0.05 ; **P ≤ 0.01; ***P ≤ 0.001.

To determine the effect in arthritis of dissociation of HK2 from mitochondria, MJ was injected either same day as K/BxN serum (d0) (**Figures 7A-C**) or 5 days (**Figures 7D, E**) in the K/BxN arthritis model, using doses of 25 and 100 mg/kg. MJ injection at day 0 served a preventative method of arthritis progression, whereas injection at day 5 demonstrated a treatment option at the peak of disease activity. Clinical score and joint diameter were significantly reduced in a dosage at 25 mg/kg over a dosage at 100mg/kg at both preventing arthritis (d0) and treating arthritis (d5). Interestingly, dosage at 100 mg/kg had increased arthritic clinical score compared with dosage at 25 mg/kg but still less than control, suggesting a reverse effect at higher doses (representative images are shown in **Figure 7C**). Histological analysis revealed synovial hypertrophy, inflammation, cartilage damage, and bone damage scores were reduced in 25 mg/kg compared with control but not in 100 mg/kg in both d0 and d5 injections but was more prominent in d0.

## Discussion

Our study looks at the role of HK2 in RA FLS and whether or not its mitochondrial binding plays a role in RA FLS behavior. Mitochondria-bound HK2 is associated with enhanced tumorigenesis in cancer, which makes this specific mitochondria-bound isoform a more attractive target (3, 49, 50).

We found that HK2 translocated to the mitochondria after PDGF stimulation, a common cytokine found in RA synovium (51). In other tissues, for instance, in human carotid artery smooth muscle cells, PDGF stimulation activated the Akt pathway, which increases HK2 expression *via* HIF-1a upregulation (52), promoting glycolysis and mitochondrial membrane potential hyperpolarization. When infected with HK2 containing the mitochondrial binding motif, RA FLSs were found to become more aggressive during functional assays such as invasion and migration. Other data in cardiomyocytes have shown that the mitochondrial binding motif that is located on the N terminus is necessary and sufficient for HK2 binding and that Akt phosphorylates HK2 at Thr-473 that increased mitochondrial binding (53).

MJ is a drug that was found to increase necrosis and apoptosis in hepatocellular carcinoma cells by detaching HK2 from a voltage-dependent anion channel and inhibited glycolysis (54, 55). Other groups found that the amount expression of HK and its binding to the mitochondria determines susceptibility to jasmonates in cancer cells and suggests selectivity to cancer cells (47). In our studies, MJ dissociates HK2 from the mitochondria in FLS and reduces migration and invasion as compared to control with stimulation with PDGF. The drug was also sufficient at reducing arthritis in the K/BxN animal model and even better at prevention of severe arthritis. Of interest, some reports already suggested a protective role of MJ in arthritis, such as *in vivo* studies on the LPS-induced arthritis murine model (56, 57). Our study found that Tofa, a common drug used in treatment of RA, but not MTX, detached HK2 from the mitochondria, suggesting that HK2 dissociation could be involved in its therapeutic effects. Another group described the effect of Tofa on the expression of glycolysis-related genes, including HK2, and its effect on RA FLS invasion (58).

In cancer, membrane-bound HK2 increases consumption of glucose and production of lactate to increase proliferation and resistance to apoptosis induced from chemotherapy (59, 60). Manipulation of an isoform-specific enzyme could prove to be a safer and more specific approach than global glycolytic inhibition to avoid unnecessary tampering with healthy tissue and cells. An even more specific strategy would be to inhibit the binding of HK2 to the mitochondria, because it appears to have a significant effect on RA FLS behavior. In cancer cells, detaching HK2 from the mitochondria also induced apoptosis by releasing cytochrome c into the cytosol and deceases the tumor size (12, 47, 61). Dissociation of the HK2 from mitochondria also modulates mitophagy and autophagy during metabolic suppression (40), yet, in other pathologies, such as prevention of ischemia–reperfusion injury, the opposite effect is desirable (62).

scRNA-seq data found that *Hk2*-positive fibroblasts correlated with several genes, some of them related to myofibroblast signatures such as the collagen alpha (Col) genes (*Cola1*, *Col3a1*, etc.) and alpha actin-2 (*Acta2*), and their expression was decreased by dissociation of mitochondrial HK2. A myofibroblast signature was described in RA FLS from a high-inflammation synovium (63, 64). Other genes like periostin were already associated with migration and invasion of synoviocytes (65). Finally, Tumor necrosis (TNF) and factor Interleukin 6(IL-6) did not decrease after MJ incubation (data no shown), which, together with the recent data that suggested HK2 to also be dispensable for T cell activation, proliferation, and differentiation (66), implies that immune system would not be compromised using this type of inhibitor (67).

In summary, our results suggest that mitochondrial HK2 is a key regulator of aggressive FLS phenotype. New therapeutic approaches to dissociate HK2 from mitochondria offer a safer approach than global glycolysis inhibition.

## Data availability statement

The original contributions presented in the study are publicly available. This data can be found here: https://www.ncbi.nlm.nih.gov/geo/ under the accession number GSE230145.

## Ethics statement

The animal study was reviewed and approved by University of California, San Diego.

## Author contributions

All authors were involved in drafting the article or revising it critically for important intellectual content, and all authors approved the final version to be published. Dr. MG had full access to all the data in the study and takes responsibility for the integrity of the data and the accuracy of the data analysis. Study conception and design: SM and MG. Acquisition of data: AT, SKa, CM, PO, MF-B, and SK. Analysis and interpretation of data. AT, SKa, CM, PO, MF-B, SK, TL, PG, AC, ES-L, SM, and MG. All authors contributed to the article and approved the submitted version.

## Supplementary material

Supplementary Figure 1
**(A)** Hexokinase activity in peripheral blood mononuclear cells (PBMCs) and RA fibroblast-like synoviocytes (FLS) before and after PDGF stimulation. **(B)** Schematic representation of adenovirus constructs including full-length (HK2FL), N terminus deletion mutant (HK2ΔN), and 15G. N-terminus mitochondrial binding site represented in purple while the HK2 enzyme is represented in green. **(C)** Protein analysis of HK2 after infection with HK2FL adenovirus, HK2ΔN adenovirus, or GFP adenovirus. **(D)** Confocal microscopy of RA FLS after infection with either GFP adenovirus, HK2FL adenovirus, and HK2ΔN adenovirus after 48 hours incubation with quantification. HK2 protein stained green, ATP5b stained red, DAPI stained blue. Yellow overlap indicates HK2 localization to mitochondria. n = 3 independent experiments. The comparison between groups were performed using unpaired two-tailed student *t*-test. Statistical significance was considered when p value ≤0.05.

Supplementary Figure 2
**(A)** MTT viability assay of RA FLS under EtOH, 1mM MJ, 1.5mM MJ, or 2.5mM MJ after 24 hours. Comparison between groups were performed using Kruskal-Wallis test followed by Dunn’s multiple comparisons test. Statistical analysis of P value ≤0.05 were considered significant. **(B)** Cytochrome C release in RA FLS (n=3) after 4-hour incubation with PBS, EtOH, and MJ 2.5mM. Comparison between groups were performed using ordinary one-way ANOVA followed by Tukey’s multiple comparisons test. Statistical analysis of P value ≤0.05 were considered significant. **(C)** Mitochondrial Fractionation. RA FLSs were inhibited with methyl jasmonate for 60 minutes and protein was separated by cytosolic (C) and mitochondrial (M) fractionation and analyzed through Western blot. GAPDH is control protein for cytosolic fraction and TOM20 is control protein for mitochondrial fraction.

Supplementary Figure 3 Confocal microscopy of RA FLS (n=3) after 48 hours incubation with HK2FL adenovirus with and without Tofacitinib 1uM with quantification. HK2 protein is stained with green fluorescence, ATP5a is stained with red fluorescence. Overlapping yellow color indicates HK2 colocalization to mitochondria. n = 4 independent experiments. The comparison between groups were performed using ordinary one-way ANOVA followed by Tukey’s multiple comparisons test. Statistical analysis of P value ≤0.05 were considered significant.

Supplementary Figure 4
**(A)** UMAP of scRNAseq data of live CD45neg cells from digested hind limbs with sample labels. CON: control, PEAK: peak, RESED: resolved, RESING: resolving. **(B)** UMAP feature plots of fibroblast markers. Violin plots of Osteoblast **(C)**, Chondrocytes **(D)**, Vascular **(E)**, Perivascular **(F)**, Muscle **(G)**, Proliferating **(H)** markers. **(I)** UMAP of fibroblast subset with sample labels. CON: control, PEAK: peak, RESED: resolved, RESING: resolving. **(J)** UMAP feature plots of fibroblast lining layer markers. **(K)** GO term dot plot from marker genes in HK2 positive compared to HK2 negative cells.
