# Supplementary figures and images for: Role of mitochondria-bound HK2 in rheumatoid arthritis fibroblast-like synoviocytes
Source: Front Immunol. 2023 Jul 17;14:1103231. doi: 10.3389/fimmu.2023.1103231 (PMC10389265; doi:10.3389/fimmu.2023.1103231)

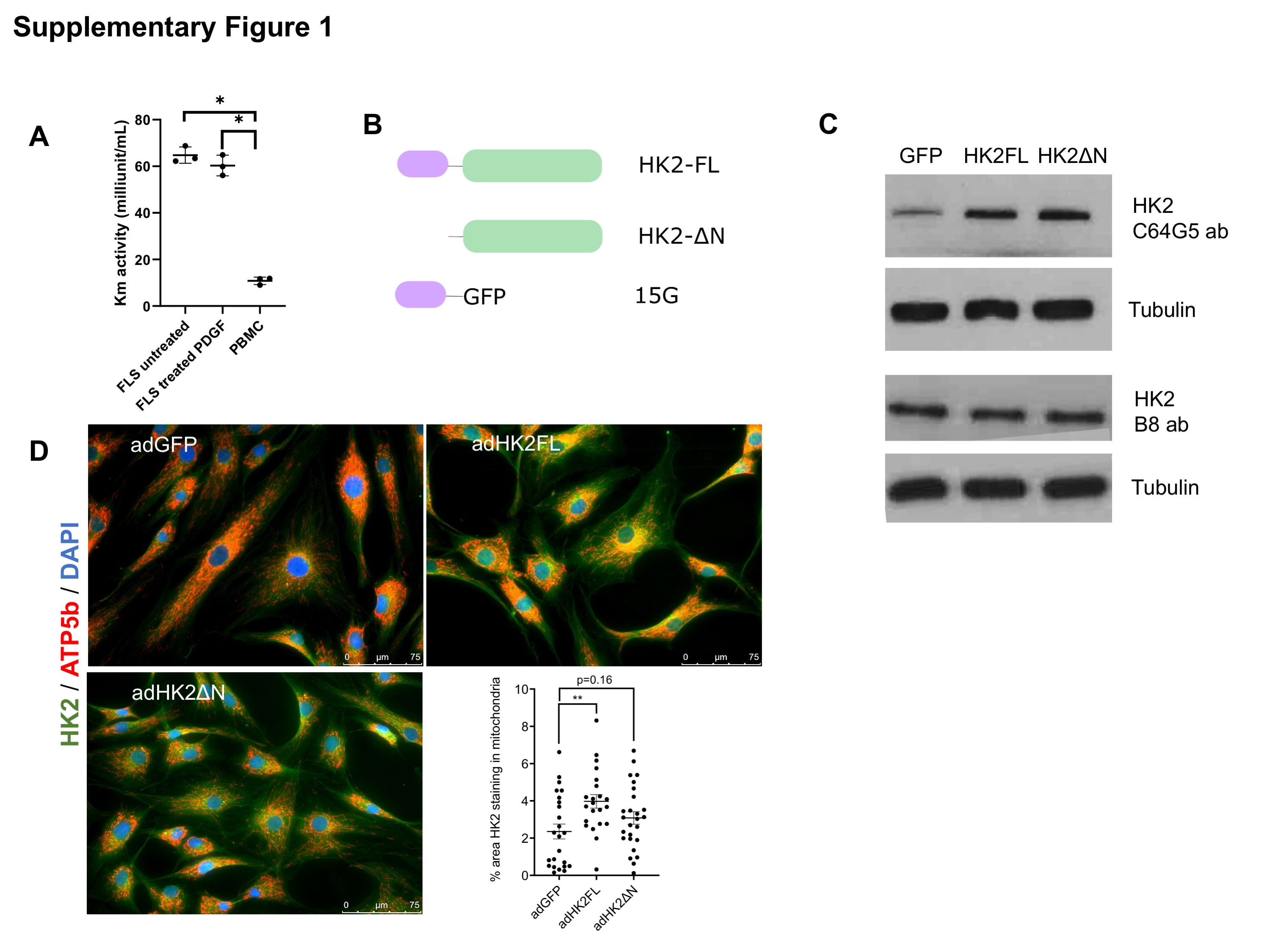

Supplement: Supplementary Figure 1 — (A) Hexokinase activity in peripheral blood mononuclear cells (PBMCs) and RA fibroblast-like synoviocytes (FLS) before and after PDGF stimulation. (B) Schematic representation of adenovirus constructs including full-length (HK2FL), N terminus deletion mutant (HK2ΔN), and 15G. N-terminus mitochondrial binding site represented in purple while the HK2 enzyme is represented in green. (C) Protein analysis of HK2 after infection with HK2FL adenovirus, HK2ΔN adenovirus, or GFP adenovirus. (D) Confocal microscopy of RA FLS after infection with either GFP adenovirus, HK2FL adenovirus, and HK2ΔN adenovirus after 48 hours incubation with quantification. HK2 protein stained green, ATP5b stained red, DAPI stained blue. Yellow overlap indicates HK2 localization to mitochondria. n = 3 independent experiments. The comparison between groups were performed using unpaired two-tailed student t-test. Statistical significance was considered when p value ≤0.05. [file Image_1.jpeg]

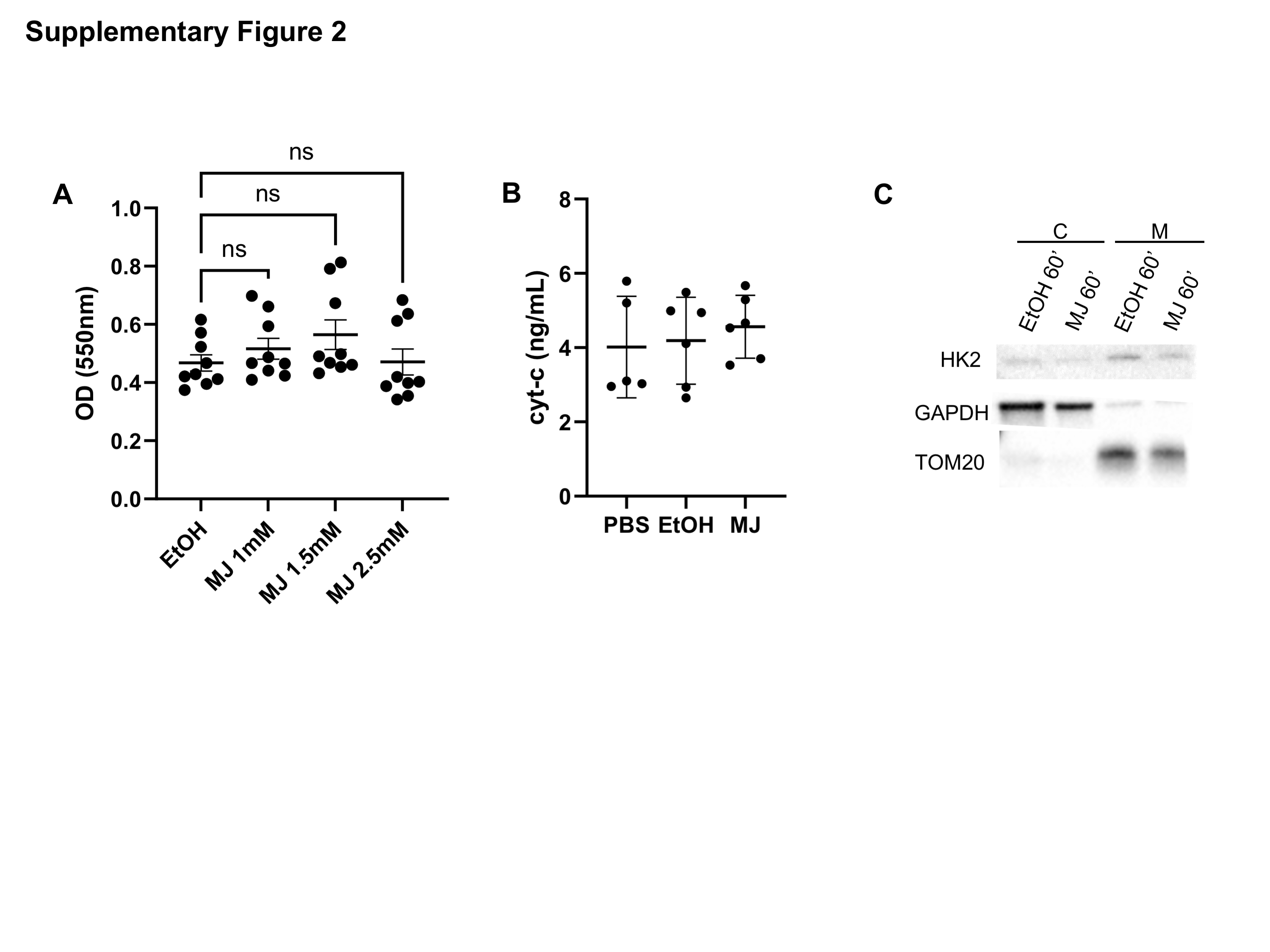

Supplement: Supplementary Figure 2 — (A) MTT viability assay of RA FLS under EtOH, 1mM MJ, 1.5mM MJ, or 2.5mM MJ after 24 hours. Comparison between groups were performed using Kruskal-Wallis test followed by Dunn’s multiple comparisons test. Statistical analysis of P value ≤0.05 were considered significant. (B) Cytochrome C release in RA FLS (n=3) after 4-hour incubation with PBS, EtOH, and MJ 2.5mM. Comparison between groups were performed using ordinary one-way ANOVA followed by Tukey’s multiple comparisons test. Statistical analysis of P value ≤0.05 were considered significant. (C) Mitochondrial Fractionation. RA FLSs were inhibited with methyl jasmonate for 60 minutes and protein was separated by cytosolic (C) and mitochondrial (M) fractionation and analyzed through Western blot. GAPDH is control protein for cytosolic fraction and TOM20 is control protein for mitochondrial fraction. [file Image_2.jpeg]

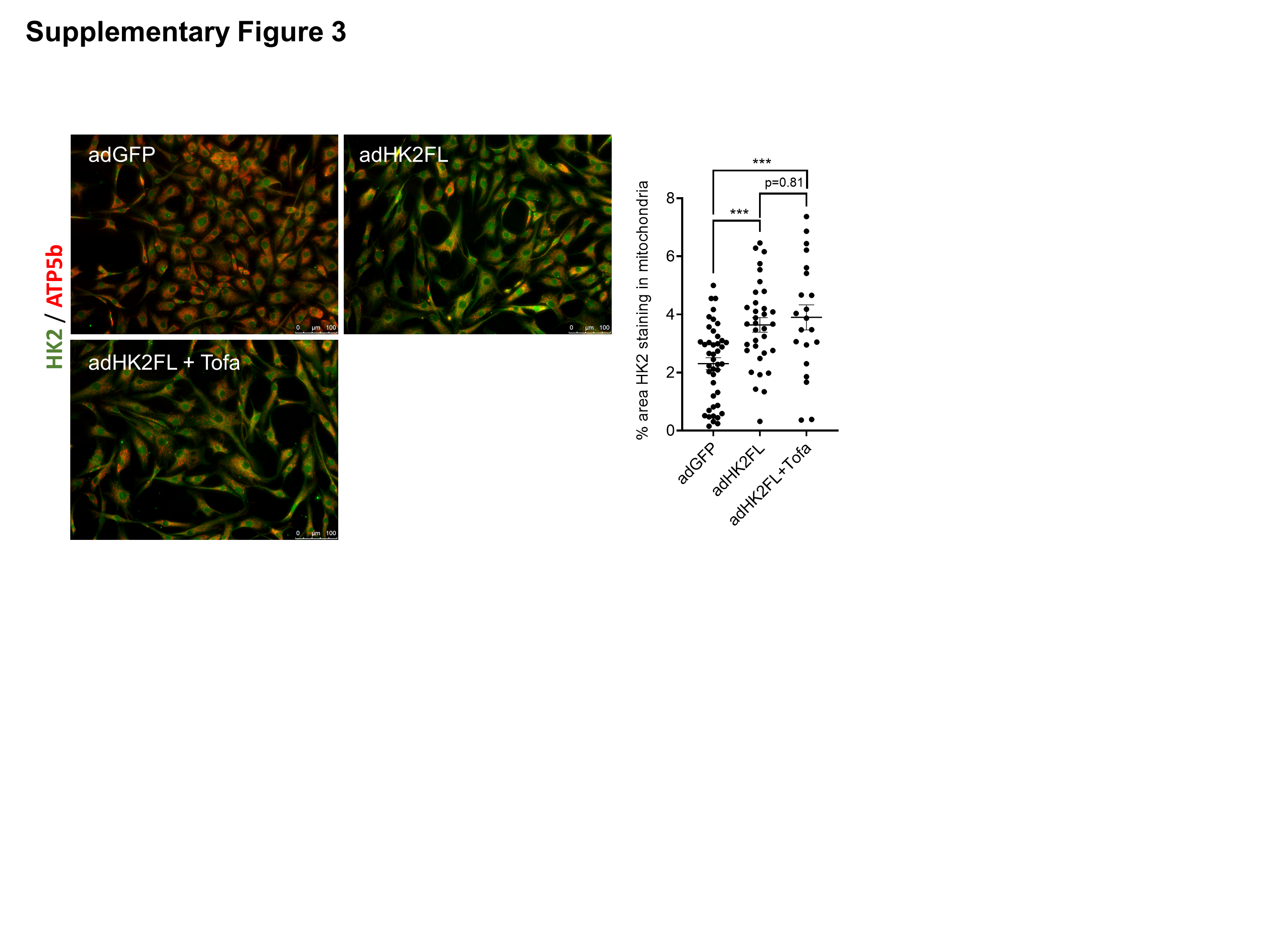

Supplement: Supplementary Figure 3 — Confocal microscopy of RA FLS (n=3) after 48 hours incubation with HK2FL adenovirus with and without Tofacitinib 1uM with quantification. HK2 protein is stained with green fluorescence, ATP5a is stained with red fluorescence. Overlapping yellow color indicates HK2 colocalization to mitochondria. n = 4 independent experiments. The comparison between groups were performed using ordinary one-way ANOVA followed by Tukey’s multiple comparisons test. Statistical analysis of P value ≤0.05 were considered significant. [file Image_3.jpeg]

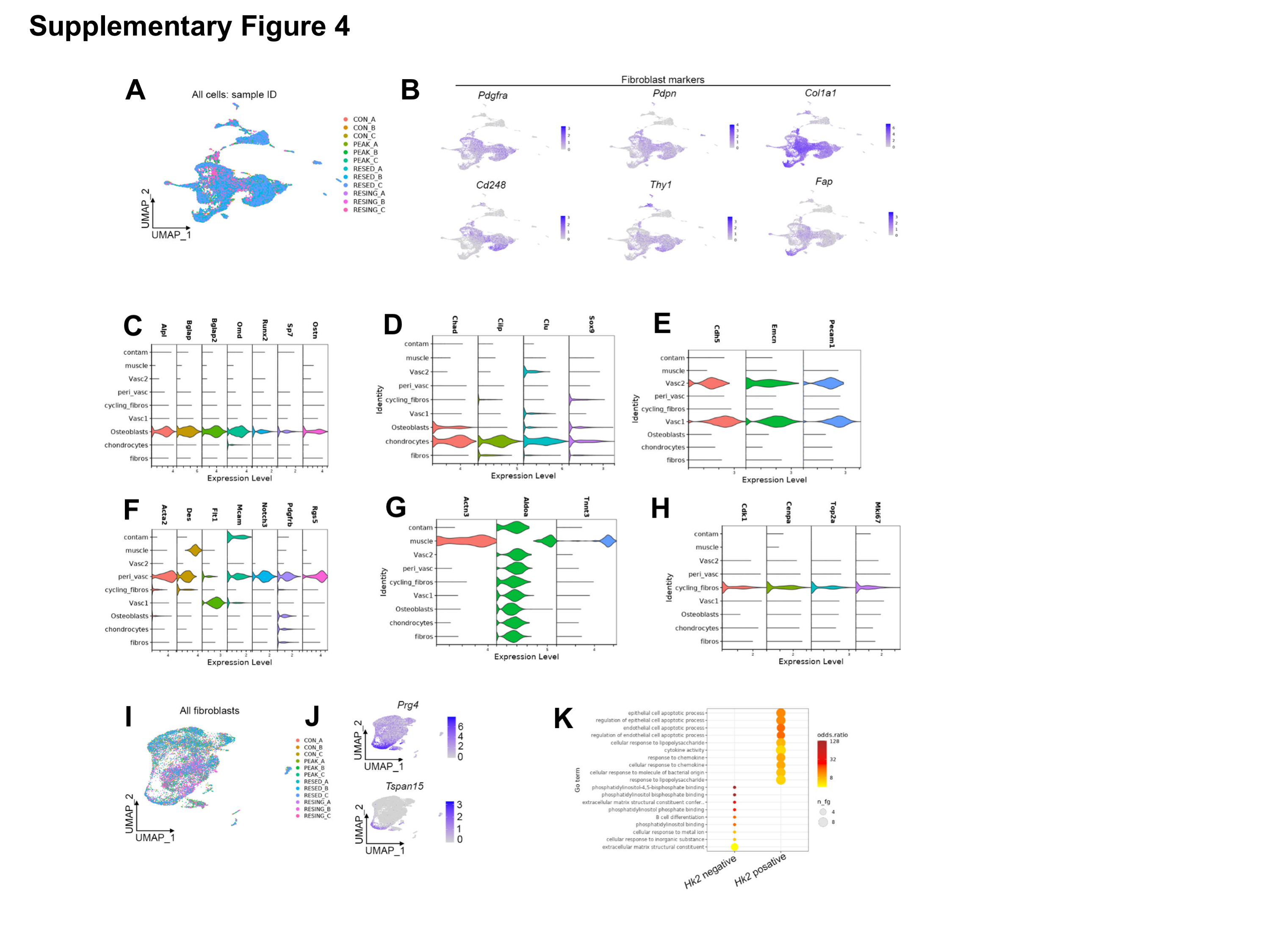

Supplement: Supplementary Figure 4 — (A) UMAP of scRNAseq data of live CD45neg cells from digested hind limbs with sample labels. CON: control, PEAK: peak, RESED: resolved, RESING: resolving. (B) UMAP feature plots of fibroblast markers. Violin plots of Osteoblast (C), Chondrocytes (D), Vascular (E), Perivascular (F), Muscle (G), Proliferating (H) markers. (I) UMAP of fibroblast subset with sample labels. CON: control, PEAK: peak, RESED: resolved, RESING: resolving. (J) UMAP feature plots of fibroblast lining layer markers. (K) GO term dot plot from marker genes in HK2 positive compared to HK2 negative cells. [file Image_4.jpeg]
